# Nurses’ attitudes towards the nursing profession and associated factors in selected public hospitals, Addis Ababa, Ethiopia, 2021: a cross-sectional study

**DOI:** 10.1186/s12912-022-00808-2

**Published:** 2022-01-17

**Authors:** Abdurkie Defa Rekisso, Zuriash Mengistu, Teshome Habte Wurjine

**Affiliations:** Department of Nursing, School of Nursing and Midwifery, College of Health Sciences, Addis Ababa University, Addis Ababa, Ethiopia

**Keywords:** Attitude, Nurses, Nursing profession, Addis Ababa

## Abstract

**Background:**

Unfavorable attitude toward nursing profession remaining the global issue across in the various level of health institutions attitude is the most important concept in nursing. Because of high ambiguity and role conflict in the profession.

**Objective:**

This study aimed to explore nurses’ attitudes toward their profession and associated factors in selected public hospitals, Addis Ababa, Ethiopia, 2021.

**Methodology:**

Institutional based quantitative cross- sectional study design was conducted in five randomly selected public hospitals of Addis Ababa, Ethiopia, from May to April 2021. Systematic random sampling method was used to select sample of 357 nurses working in selected hospitals. Data were collected using standard self-administer questionnaire and the collected data were entered and analyzed by using SPSS 25version. Descriptive, Bi-variate and multiple logistic regression analysis were computed to describe the association between attitude of nurses and independent variables that show *P*-values ≤0.05 with 95% Confidence Interval consider statistically significant factors for attitude toward professionalism.

**Results:**

A total of 348 nurses were participated in the study, with 97.5% response rate. From this 60.6% (*n* = 211) of study participants were female nurses. Only 46% of nurses in Addis Ababa city public hospitals had favorable attitude towards their profession. Experienced nurses [(AOR: 1.19; 95% CI: 0.625, 13.37)], had good managerial support [(AOR: 2.40; 95% CI: 0.197, 26.702)], had ethical related training [(AOR: 1.50; 95% CI: 0.35, 6.407)], had positive image toward nursing profession [(AOR: 2.32; 95% CI: 0.166, 34.950)], who believe in our community had positive image toward nursing profession [(AOR: 4.73; 95% CI: 2.136, 88.109)] were positively associated with the overall nurses’ attitude toward their profession.

**Conclusion:**

This study offers an interesting insight about nurses’ image toward their profession and associated factors in Addis Ababa city. The overall attitude of nurses in Addis Ababa city public hospitals toward their profession was falls below the average level. Given the importance of the attitude in nursing and various factors, efforts are directed to achieve the desired level and reducing the barriers.

## Introduction

### Background information

Nurses are the largest group of employees who provide health care services and have a crucial role in the realization of an effective health-services, in any health care setting [1]. For many years, started in Florence nightingales nursing has been denied as a profession due to the historic background of the profession and considered as physician’s assistant in the work place [2]. Even though a nurse occupies a large portion of health care professionals, they had a limited role in health-related policy and decision making activities [3]. Many nursing theorists defined nursing in many ways and Virginia Henderson (1958) is one of the nursing theorists who defined nursing as “the unique function of the nurse is to assist the individual, sick or well, in the performance of those activities contributing to health or its recovery or to peaceful death” [4]. Even though nursing is defined in a clear way still today, there are different attitudes toward nursing profession. Attitude is an idea, belief, or image formed as a result of how you perceive or comprehend something [2]. Through many centuries of struggle, the nursing profession gradually developed from a mere job to a recognized profession. Various studies conducted around the world revealed that nurses’ attitude toward their profession on different degrees. A study conducted on the nurses’ attitude towards the nursing profession about 346 nurses in Saudi Arabia reveal that 33.2% of nurses had a favorable attitude towards nursing profession 5. And another descriptive cross-sectional study design was employed to assess the nurses’ attitudes and associated factors in southern Turkey shown that 80.6% of them were not members of any nurses’ union and chosen their profession was by their family members to be a nurse [5].

Because of the high ambiguity and role conflict in the nursing profession, attitude is the most important concept [6]. Current research indicates that attitude towards their profession, nurses have strong association with nursing services and patient satisfaction [7]**.** Nurses with a positive attitude are expected to provide altruistic service, compassionate care for health customers, to be proud of their profession and able to hold intra and extra professional factors. On the other hand, nurses had negative attitude towards the nursing profession, loses their interest to serve the patients, that leads to negative outcomes like providing uncompassionated care, feeling shy, intending to resign from their profession [8]. Research done in Sudaria by the year 2020 showed that, about three-fourth of the respondents would be ashamed if they had a nurse in their family [9]. Research done in Ethiopia on assessment of nurses’ attitude and its associated factors among 332 nurses in Jimma zone public hospitals, south west Ethiopia showed that only 30.3% of nurses had a favorable attitude toward the nursing profession [10].

Nurses often havea negative perception such as “I would leave the nursing profession,” “nurses are not unified,” “what is my job,” “what I gained from this profession,” “nurses lack autonomy,” and other derogatory statements [11]. This negative image towards the nursing profession is not innate behavior and it’s emerged from multi-dimensional factors. If these factors are not addressed and resolved, we may lose a large number of employees, resulting in the nurse’s shortage and ultimately a health-care system failure might be happened. To address these problems, nurses’ managers and other-stakeholders must develop a certain strategy to over-come the negative image of nurses towards their profession.

The purpose of this study is to explore the level of nurses’ attitudes toward their profession and associated factors at public hospitals in Addis Ababa city, Ethiopia, 2021. The results of this study are significant to increase the level of nurses’ attitude toward nursing profession. Administrative managers of each hospital will apply for evidence-based process; training purpose, to take corrective measures on the identified predictors and to design an appropriate strategy to create a favorable attitude among nursing staffs. Again, the findings of the study will encourage the researchers and other stake-holders to provide as base line information to conduct a large-scale study in the area.

### Hypothesis/questions

What is attitude of nurses towards their profession?

Is there an association between nurses’ attitude and its predictors?

### Conceptual framework

**Fig. 1:**
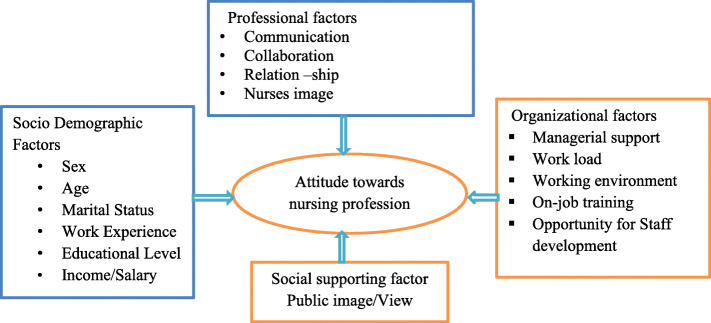
Conceptual frame-work on factors associated with nurses’ attitude toward nursing profession among nurses in selected public hospitals of Addis Ababa, Ethiopia

This conceptual framework shows relationship between dependent and independent variable of nurses’ attitude toward nursing profession and associated factors as *shown in* Fig. 1*below*. This frame-work was developed after reviewing different literature and adopted towards the sociodemographic status of the study population [7, 10].

## Methods and materials

### Study setting

The study was conducted at selected public hospitals of Addis Ababa city, Ethiopia. Addis Ababa is the largest and the most populated capital city of Ethiopia. The Addis Ababa metropolitan area with a population was estimated to be around 5,00,6000 people in 2021, This capital city holds 527 km [2] of area in Ethiopia. The population density is estimated to be near 5165 individuals per square kilometer available. Based on the 2020 population enumeration annual growth rate is 4.42% [12]. The city has 15 public hospitals from this five of them randomly selected to conduct this research study that includes: Tikur Anbessa Specialized Hospital, St. Peter’s specialized hospital, Zewditu memorial hospital, Yekatit 12 hospital and Menelik II hospital. Tikur anbessa specialized hospital is one of tertiary hospital in the country and 800 beds hospital and currently 800 nurses working and approximately 370,000–400,000 patients per year. (TASH human resource office 2021). Zewditu memorial hospital is a teaching and general hospital with 175 beds and 280 nurses. Menilik II referral has 376 nurses. Yekatit 12 hospital is 272 bed hospital with 480 nurses. And St. Peter’s specialized hospital is 271 bed hospital and 267 staff nurses (Human resource office documents 2021).

### Study design and period

An institution based-quantitative cross-sectional study design was conducted among nurses in Addis Ababa city of five randomly selected public hospitals from May to April 2021.

### Sampling technique and procedure

The sample size was calculated using a simple population proportion formula based on the assumptions 95% confidence level, 5% marginal error [13], and 30.3% population proportion [10]. The sample size was determined ***n = 357*** with considering 10% of non-response rate. Study participants were recruited using a systematic random sampling method and drawn every 6th interval of the study population of sampling frame. The number of nurses participating in the study was proportionally allocated from nurses working in the study hospitals of Addis Ababa city and systematic random sampling techniques were applied to select the study participants who met the eligibility criteria.

Five BSC nurses involved as data collectors and one MSc nurse supervisor were included in the data collection process. Training was given to the data collectors on the purpose of the study, process of data collection to evaluate the methods and materials and close supervision was made during data collection period by the assigned supervisors.

### Study population

All nurses currently working in the five randomly selected hospitals of Addis Ababa city administration, and from this sample of nurses selected to be included under study.

### Eligibility criteria

All Nurses working in the selected hospitals and who have more than six-months work experience and nurses who were on duty during data collection period.

### Operational definition

- ***Favorable attitude:*** – those who were scored greater than or equal to mean value.
- ***Unfavorable attitude:*** – those who were scored score less than mean value.
- ***Associated factors:*** - the variables which can affect nurses’ attitude toward their profession [14].

### Measurements

The data was collected using a questionnaire which was developed in English language and adapted from previously conducted studies in Al Balqa Applied University of Jordan and south west Ethiopia [14, 15]. The questionnaire focused on the nurses’ attitude toward their own profession and associated factors. The questionnaire comprised structured questions focusing on two main areas, namely: view of nurses toward their own profession and its predictors. The data collection instruments include three major parts:

- **Part I**: This section includes questions related to sociodemographic information of the study subjects such as sex, age, marital status, educational status, monthly income and work experience.
- **Part II:** It includes questionaries used to explore nurses’ image toward their own profession. This section contains 18 items with a Likert scale of five options. The participants were administered to give their opinion on each item using a 5-point Likert scale ranging from strongly disagree (1) to strongly agree (5). This questionnaire has been used for many years for assessment of nurses’ attitudes toward their own profession and both validity and reliability of the tool have been confirmed.

Tool validity and reliability for this study was estimated again for study area. To confirm the validity, the questionnaire was given to 18 nurses at Ghandi memorial hospital under Addis Ababa city administration. After some revisions were made and the questionnaire’s internal consistency was ensured with Cronbach’s alpha coefficient of 83%. The reliability was estimated to be 85%, using test-retest method with an interval of two weeks before actual data collections [5, 16, 17].

- **Part III**: This part included questions related factors associated with nurses’ perception toward their profession. It contains three dimensions, namely; organizational related factors, social supporting factors and health professional related factors questionaries.

Participants who were scored greater than or equal to the mean in the attitude test questionnaire were classified as having favorable attitude towards nursing profession and those who had scored less than the mean value was declared to having unfavorable attitude toward nursing profession [14].

### Data processing and analysis

Data was checked for completeness and consistency and then entered into statistical package for social science (SPSS) 25 for analysis. The descriptive data were presented by using frequency, percentage, mean and SD. Bivariate logistic regression with 95% CI was used to see the existence of an association between nurses’ attitudes toward their profession and its predictors separately. Then, all variables with *p*-values less than 0.2 were transferred to multiple logistic regressions analysis. Finally, a variable shows *p*-value ≤0.05 with 95% CI was taken as statically significant. Hosmer Lemenshow goodness of fit and multicollinearity among independent variables were checked.

### Data quality assurance

A structured questionnaire was used to collect the data after the instrument was pretested on 5% (18 nurses) of total sample size of study participants who were working in Gandi Memorial hospital. The pretest was conducted two weeks before the actual data collection period and the pretested data were not included the main study and training was provided for supervisors and data collectors on the data collection instruments and the gap of the method and materials were identified and appropriate correction was made.

## Results

### Socio demographic characteristics of the respondents

A total of 348 nurses participated with 97.5% of response rate. From a total participant 60.6% of them were female. A larger proportion of the study respondents, 37.9% were in the age group of 26–30 years of old, 28.7%, were in the age group of 31–35 and 22.7% were in the age group of 36–40. Educational level status, the majority of the respondents, 71% were BSc degree holders, 20.8% were master’s degrees the remaining 4.6% were Diploma holders. Marital status of the participants 57.8% were married and the remaining 42.2% were single. In terms of professional working experience, 56.6% had six-ten years of work experience, 31.3% of them had less than five years of work experience and about 12% of participants had more than ten years of work experience. The assessment result reveal that economic characteristics of respondents were 51.4% monthly salaries ranged from 7000 to 9999 ETB, 42.0% earned 4000–6999 ETB and only 6.6% earned more than 10,000 ETB per month.

### Attitude toward nursing profession

Attitude toward nursing profession was tested by 18 questionaries which was obtained from Halls’ Professionalism Inventory guideline. The mean and SD of respondents were 2.92 and 0.29 respectively. As *depicted in* Table 1, the score above the mean of respondents indicates had a favorable attitude toward nursing profession and the score below the mean had an unfavorable attitude toward nursing profession.

**Table 1:** Mean and standard deviation of attitude test questionaries

Questioners (characteristics)	Mean	SD
Respectful profession	3.78	1.02
A well appreciated profession in the society	2.69	1.07
A Women’s profession	2.39	1.03
Similar to that of the servants’ job.	2.45	1.02
An occupation and not a profession	1.69	0.93
A prestigious profession	3.56	1.08
A creative profession	3.37	1.01
An extremely hard profession that does not receive enough appreciation	3.79	1.25
An essential profession in any society	3.77	1.00
Nursing is a human profession	3.88	1.20
An independent profession by which nurses make decisions for themselves	3.51	1.12
Provide self-actualization	2.90	1.23
Nurses obey doctors’ orders without questioning them.	2.72	1.18
A. Nursing is a holy profession	3.01	1.00
Nurses waste a lot of time being busy doing nothing.	1.95	1.21
I would like my child to become a nurse	2.27	1.07
Easily anyone could be a nurse	2.29	1.00
Actually, equal to other professions	2.59	1.29

Key: *SD* Standard Deviation

**Fig. 2:**
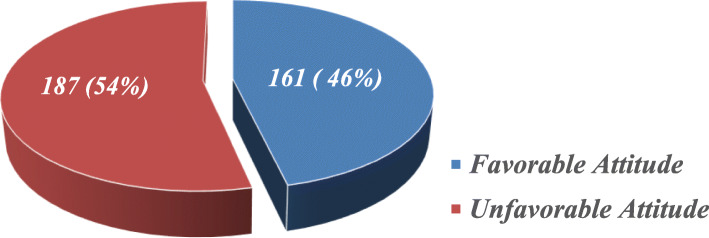
Nurses’ Attitudes toward nursing profession in selected public hospitals, Addis Ababa, Ethiopia, 2021 (*n* **=** 348)

From a total of study participants only 161 (46.3%) of them had favorable attitude toward their profession as *depicted in* Fig. 2*below*.

The study result reveal that, question was asked on nurses’ feelings about their profession, 103 (29.4%) were responded as proud to be a nurse and like their profession whereas, the remaining 248 (71%) of them had a negative attitude towards their profession.

This study indicates that, only 127 (36.5%) of study participants were members of Ethiopian Nursing Association whereas majority of the participants 63.5% were not a member of the Ethiopian nursing association and their justification for not a member of Ethiopian Nursing Association 124 (56.1%) of them responded as the association does not stand for the rights of the nurses, The nursing profession have no autonomy for their profession. In addition to this, 225 (64.7%) of the study participants claimed as they were intended to resign from their profession due to less amount of monthly salary and fear of professional risk which accounts 141(62.7%) and 38(16.9%) respectively.

### Factors affecting professional attitude

According to this study, factors affecting nurses’ attitude toward professional nursing were classified into four categories. Those factors were socio-demographic factors, organizational related factors, social supporting factors and health professional related factors. Binary logistic regression was computed to see the existence of an association between nurses’ attitude toward their profession and each independent variable separately. The variables showed P- value of less than 0.2 were transferred to multiple logistic regression analysis. Finally, a variable shows *p*-value ≤0.05 with 95% CI was declared as statically significant.

### Socio-demographic factors

For multivariable analysis variables such as; marital status, monthly income, age of respondents, gender, educational level and work experience were candidate and entered for analysis. Among those variables under socio demographic factors only, monthly income and age of respondents were showed statically significant. Study result revealed that, older age nurses were 1.19 times more likely to have a favorable toward nursing profession than younger age group [(AOR: 1.19; 95% CI: 0.625, 13.37)] *as shown in* Table 2*below*. Another variable shows significant association in multivariate analysis was monthly income.

**Table 2:** Bivariate and Multivariate analysis of socio demographic factors and attitude toward nursing profession in Addis Ababa selected public hospitals, Ethiopia 2021

Independent Variables	Categories	Attitudes		COR	AOR	Cl.of AOR	*P*-value
		Fav	Unfav				
Sex of respondent	Male	53	84	1	1		
	Female	108	103	0.51	0.330	0.017–5.407	0.634
Age of respondent	Younger (≤40 yrs)	96	134	1	1		
	Older (> 40 yrs)	65	53	2.78	**1.19**	0.625–13.37	0.005*
Monthly salary	Less paid	49	123	1	1		
	Better paid	112	64	4.19	**3.25**	1.873–68.226	0.000**
Work experience	Shorter	123	179	1	1		
	Longer	38	8	1.30	0.879	0.243–4.125	0.519
Educational status	Diploma	7	9	1	1		
	BSc and above	159	173	1.80	0.274	0.002–3.347	0.554
Marital status	Single	48	99	1	1		
	Married	113	88	0.97	0.518	0.166–3.075	0.536

Nurses who earned a better salary were 3 times more likely to have a favorable attitude toward professional nursing than those who paid less salary [(AOR: 3.25; 95% CI: 1.873, 68.226)].

### Health professional related and social supporting factors

For multivariable analysis variables such as; nurses’ image toward their profession, communication between nurses and physicians, relationship between nurses and other health team and public image towards the nursing profession were assessed. Variables such as; nurses’ image toward their profession and public image were showed significant association with nurses’ attitude toward their profession. Nurses who had a positive image in their profession were 2 times more likely to have a favorable attitude toward nursing profession than nurses who had a negative image in their profession [(AOR: 2.32; 95% CI: 0.166, 34.950)] *as depicted in* Table 3*below*. Again, Nurses who agreed with the statement “our community has a positive image towards nursing profession” were 5times more likely to have a favorable attitude for professional nurses than nurses who disagreed with the statement [(AOR: 4.73; 95% CI: 2.136, 88.109)].

**Table 3:** Bivariate and Multivariate analysis of health professional and social supporting factors and attitude toward nursing profession in Addis Ababa selected public hospitals, Ethiopia 2021

Variables	Categories	Attitudes		COR	AOR	Cl.of AOR	*P*-value
		Fav	Unfav				
Self-image	Negative	80	146	1	1		
	Positive	81	41	3.605	**2.32**	0.166–34.950	0.003**
Public image	Negative	13	154	1	1		
	Positive	148	33	3.973	**4.73**	2.136–88.109	0.000^**^
Relationship within health team	Poor	38	95	1	1		
	Good	123	92	1.207	0.609	0.170–5.390	0.937
Communication within physician	Poor	59	111	1	1		
	Good	102	76	0.971	0.308	0.065–1.384	0.124

### Organizational related factors

For multivariable analysis variable such as availability of good managerial support, availability of opportunity for staff development and on-job ethical related training were shows significant association with nurses’ attitude toward nursing profession. Nurses who agreed with the availability of good managerial support in their institutions were 2 times more likely to have a favorable attitude towards their profession than those who disagreed with the statement [(AOR: 2.40; 95% CI: 0.197, 26.702)] *as shown in* Table 4*below*. Again, Nurses who agreed with the availability of opportunity for staff development at their institution were 1.37times more likely had a favorable attitude toward nursing profession as compared with counterpart [(AOR: 1.37; 95% CI: 0.123, 5.884)] and availability of on-job ethical related training [(AOR: 1.50; 95% CI: 0.35, 6.407)].

**Table 4:** Bivariate and Multivariate analysis of organizational related factors and nurses’ attitude toward their profession in selected public hospitals, Addis Ababa, Ethiopia 2021

Variables	Categories	Attitudes		COR	AOR	Cl. of AOR	*P*-value
		Favorable	Unfavorable				
Working environment	Not conducive	62	108	1	1		
	Conducive	99	79	0.78	0.014	0.003–2.478	0.985
Managerial support	Poor	60	158	1	1		
	Good	101	29	2.61	**2.40**	0.197–26.702	0.001**
Opportunity for staff development	Not available	53	134	1	1		
	Available	112	49	1.86	**1.37**	0.123–5.884	0.028*
Ethical related training	Not available	52	109	1	1		
	Available	97	90	2.99	**1.50**	0.35–6.407	0.012**

## Discussion

The aim of this study was to determine the attitudes of nurses toward their profession and its predictors. The current study reveals that, 46% of study participants had a favorable attitude toward their profession. This result is lower than a study done in Arsi zonal public hospitals that indicates 58.7% [7]. This difference may be due to different sociodemographic characteristics and the application of data collection instrument. In the contrary, this finding is higher than a study conducted at public hospitals in South West Ethiopia 30.3% [10]. The possible reason behind is may be related to the existence of continual education opportunities in my study area than those who were working in public hospitals in south west Ethiopia.

Again, the overall percentage of nurses believed in “nursing is a well appreciated profession in society” was 37.6% (*n* = 131). This study result is lower than study conducted in Nigeria at tertiary hospitals in Enugu, 61.1%(*n* = 165) [11]. This difference might be due to study setting. Really, our communities perceive this profession as a physician’s assistant and unable to do any task independently. Besides this, study participants reported that only 29.6%(*n* = 103) felt proud and 14.1% (*n* = 49) felt shy. This study result is much lower than the study conducted in Jeddy, which showed that 59.1% of them felt proud and 6.9% felt shy [18]. These differences might be due to low payment, low health facilities and the absence of job description in our setup as compared to nurses in Jeddy. According to the findings of this study, monthly income was significantly associated with nurses’ attitude toward nursing profession. According to study result nurses who earned a better salary were 3 times more likely to have a favorable attitude towards their profession as compared to those nurses who earned less salary. This study result is in line with study done in West Shewa zonal public hospitals [19]. In fact, monthly income determines our attitude and the service we provide.

Similarly, this study result showed that older age nurses were 1.19 times more likely to have a favorable attitude towards their profession nursing than other age groups. This study result is parallel with research done in Southern-Turkey [5]. In fact, as you advance in your career, you may face numerous challenges. This difficulty tests you and makes you a good professional. Good nurses expected to have favorable attitude towards nursing profession. Managerial support shows, significant association with nurses’ attitude. This study result showed that, nurses who had good managerial support in their institution were 2 times more likely to have a favorable attitude toward professional nursing than those who had poor managerial support. This result is similar with study done in Mekelle public hospitals [20]. A compassionate leader will encourage you to have a favorable attitude and will assist you in providing qualified services. Study result revealed that, nurses’ image toward their profession was significantly associated with nurses’ attitude toward nursing profession. Nurses who had a positive image toward their profession were twice more likely to have a favorable attitude toward professional nursing than nurses who had a negative image towards their profession. This is congruent with study done in Jimma town public hospitals [15]. In-fact we manifest what is inside of us. Those who believe in positive were considering themselves as a cost-benefit in a health care setting.

Besides, the public image toward professional nursing was significantly associated with nurses’ attitude. According to a study report, nurses who agreed with the statement “our community has a positive image towards our profession” were 5 times more likely to have a positive attitude toward professional nursing than nurses who disagreed with the statement. This is similar to the study conducted in Egypt [16].

In-fact, people in the world has poor perception toward nursing profession due to historical back-ground of nursing [17]. The study result revealed that nurses who had updated on-job ethical related training were about 2 times more likely to have a favorable attitude toward professional nursing than those who had no training. This is consistent with research conducted in Iran [21]. Having ethical related training and the availability of continual educational opportunity makes them to learn new knowledge to motivate them and internalize to apply in their profession. The finding indicates that Hall’s professional nurses attitude their day-to-day clinical practice are highly important to bring clinical service quality.

### Limitation of the study

One limitation could be noted from the finding of this study was the use a single quantitative approach. A quantitative approach may not explore detailed and hidden information. It is recommended to apply a mixed methods that can be very helpful in assessing nurses’ attitudes towards their profession and associated factors. And noted a cross-sectional study design with small sample size limits generalized.

## Conclusion

The findings give us more emphasis on level of nurses’ attitude toward their own profession and associated factors among nurses in Addis Ababa city public hospitals. Favorable attitude toward their own profession leads to professional satisfaction and provision of better services to the clients. The results show that the prevalence of nurses’ attitude toward nursing profession was falls below the average level. It can be stated that with respect to professional conduct, nurses are far from the desirable state. The major point here is that, nurses at least contribute their role to become true nurses.

## Recommendations

- The Ethiopian Nursing Association, as well as other stakeholders should work hard to raise community awareness of nursing profession and contribute their role in development of this profession through various media.
- Human resource office and CEOs of each hospital should develop different ethical related training programs for nurses and open for continual educational opportunity.
- Hospital managers should give consistent support and guidance to enhance the level of nurse attitude toward their profession at their institutions.
- Ethiopian government and civil service should conduct national survey on the fairness of salary of nurses by considering work burden and role of nursing in health care services and give corrective action accordingly.
- Further investigation should be conducted to identify problems of nurses and nursing.

## Data Availability

All relevant data are included with in the manuscript document. If it is necessary, it is possible to contact the corresponding author to get additional materials.

## Abbreviations

AAU: Addis Ababa University
ANA: America nursing association
AOR: Adjusted odd-ratio
CHS: College of health science
COR: Crude odd- ratio
CSA: Central statistical agency
DC: Data collector
ETB: Ethiopian Birr
FMOH: Federal Minister of Health
SPSS: Statistical package for social science
VIF: Variable inflation factors
